# Gonorrhœa

**Published:** 1846-03

**Authors:** 


*Gonorrhœa.*—When is a patient secure against communicating the disease? Few practitionersof experience but have had questions addressed to them with earnestness on this subject. There are cases in whi h it is of great importance to be able to give a definite answer to such questions. We will quote Ricord's opinion on this topic, and the quotation will also embrace his views of the conduct which the medical adviser is bound to pursue in certain cases of peculiar delicacy and responsibility. Mr. Acton says, "Ricord considers that when the secretion is reduced to a thready mucus, which is transparent, like vermicelli, contagion is not to be dreaded. On the contrary, so long as the secretion is purulent, it is capable of causing a similar complaint in any mucus membrane with which it comes in contact." * * * * "Patients sometimes present themselves to me to know if they may marry. I persuade them against it if they have a simple gonorrhœa; but if it be a virulent complaint,—i. e. Syphilitic—I wash my hands completely of the affair. If they still persist, I tell them that they may give a gonorrhœa to their wives, which, unless cured previously to confinement, may cause a loss of eyesight to the child:—called, however, afterward to cure the lady, I attempt to explain theaffection which she has contracted, by speaking of the fatigue of the honey moon, as well as the *dejeuner a la fourchette,* and, in the interim, cure both parties. Such is the part that a medical man often has to play, and many disputes in married life, may be thus avoided, and the Surgeon must lend himself to deception."—*Ricord on Venereal Diseases.*

